# Polymorphisms of *FDPS*, *LRP5*, *SOST* and *VKORC1* genes and their relation with osteoporosis in postmenopausal Romanian women

**DOI:** 10.1371/journal.pone.0225776

**Published:** 2019-11-27

**Authors:** Alina Deniza Ciubean, Rodica Ana Ungur, Laszlo Irsay, Viorela Mihaela Ciortea, Ileana Monica Borda, Gabriela Bombonica Dogaru, Adrian Pavel Trifa, Stefan Cristian Vesa, Anca Dana Buzoianu

**Affiliations:** 1 Department of Rehabilitation, University of Medicine and Pharmacy “Iuliu Hațieganu”, Cluj-Napoca, Romania; 2 Department of Genetics, University of Medicine and Pharmacy “Iuliu Hațieganu”, Cluj-Napoca, Romania; 3 Department of Pharmacology, Toxicology and Clinical Pharmacology, University of Medicine and Pharmacy “Iuliu Hațieganu”, Cluj-Napoca, Romania; Charles P. Darby Children’s Research Institute, UNITED STATES

## Abstract

**Objectives:**

This study aimed to assess the relationship between bone mineral density and genotypes of four polymorphisms in previously detected osteoporosis-candidate genes (*FDPS* rs2297480, *LRP5* rs3736228, *SOST* rs1234612, *VKORC1* rs9934438) in postmenopausal Romanian women with primary osteoporosis.

**Methods:**

An analytical, prospective, transversal, observational, case-control study on 364 postmenopausal Romanian women was carried out between June 2016 and August 2017 in Cluj Napoca, Romania. Clinical data and blood samples were collected from all study participants. Four polymorphisms were genotyped using TaqMan SNP Genotyping assays, run on a QuantStudio 3 real-time PCR machine.

**Results:**

Women with TT genotype in *FDPS* rs2297480 had significantly lower bone mineral density values in the lumbar spine and total hip, and the presence of the T allele was significantly associated with the osteoporosis. Women carrying the CC genotype in *LRP5* rs3736228 tend to have lower bone mineral density values in the femoral neck and total hip. No significant association was found for the genotypes of *SOST* rs1234612 or *VKORC1* rs9934438.

**Conclusions:**

Our study showed a strong association between bone mineral density and polymorphisms in the *FDPS* gene, and a borderline association with *LRP5* and *SOST* polymorphisms in postmenopausal Romanian women with osteoporosis. No association was found for *VKORC1*.

## Introduction

Osteoporosis (OP) is a common and complex skeletal disorder characterized by decreased bone mass and bone quality, which lead to an increase vulnerability to fragility fractures [1]. From a genetic perspective, the etiology of OP and fracture risk susceptibility is multifactorial, involving significant environmental influence together with genetic factors across numerous biologic processes, and it is thought that 60% to 80% of bone loss acceleration is due to genetic factors. [2]

More than 66 bone mineral density (BMD) loci have been studied in genome wide association studies (GWASs), confirming the highly polygenic nature of BMD variation. Although over the past decades there has been significant progress in identifying candidate genes involved in BMD, fracture and other related phenotypes, most of the genetic variants remain to be uncovered or validated in various ethnic groups.

To date, multiple single nucleotide polymorphisms (SNPs) in several genes have been associated to BMD, but the results are inconclusive and conflicting. As OP is a polygenic disease, and each bone phenotype (density, quality, metabolic rate) is the result of interaction among multiple genes, and the “essential” one, responsible for OP, is not yet identified despite using advanced methods [3,4].

One of the most important signaling pathways in bone in Wnt, which is crucial for bone development during embryogenesis and has a dual role in bone mass regulation, influencing both bone formation and resorption. The components of the Wnt pathway are proteins involved in cell proliferation, differentiation, and apoptosis of bone cells.[5] When cells are stimulated through the membrane receptors low-density lipoprotein receptor-related protein 5/6 (LRP5/6), the architecture of the multiprotein complex is modified, which inhibits b-catenin, leading to its translocation to the nucleus, where it initiates the transcription of target genes.[6] *LRP5* is the most important membrane receptor of the Wnt signaling pathway and it was previously tagged in a genome-wide association study to be associated with OP.[7] *LRP5* inactivation caused by mutation is responsible for osteoporosis-pseudoglioma syndrome, in which low bone mass and fractures occur.[8] Also, there are several naturally occurring inhibitors of Wnt signaling, such as Dickkopf (DKK) and sclerostin (SOST) proteins that inactivate signaling from LRP5/6 receptors. Sclerostin, encoded by the SOST gene, antagonizes Wnt signaling in both osteocytes and osteoblasts by binding to the LRP5/6 coreceptor and preventing bone formation.[9,10] High-bone-mass syndromes are thought to be caused by inactivating mutations of SOST (sclerosteosis and van Buchem’s disease).[11] Animal studies have indicated that sclerostin inhibition increases bone mass by stimulating bone formation and inhibiting bone resorption.[12]

The discovery of mevalonate pathway’s role as the target of the antiresorbtive agents from the amino-bisphosphonates (N-BP) class, used in postmenopausal OP treatment, revealed other important genes to be considered as candidate genes, like farnesyl diphosphate synthase (*FDPS*) or geranylgeranyl pyrophosphate synthase (*GGPS1*), which maintain the resorption activity of the osteoclasts. *FDPS* is a key-enzyme of the mevalonate pathway and is a well recognised target of several N-BPs, making it worthy of being studied. [13–19]

Also, even though its hemostatic effect and implication in warfarin sensitivity are well known, there has been evidence that vitamin K also plays an important role in maintaining bone strength and that mutations in the vitamin K epoxy reductase (*VKORC1*) gene may modify the gamma-carboxylation of osteocalcin and may influence BMD [20–22].

Today, it is well established that OP is a multifactorial complex disorder, whose pathogenesis is due to the interaction of various genetic determinants regulating bone and mineral metabolism with “non-skeletal” risk factors that could influence fall risk (e.g. muscle strength, balance, visual acuity), environmental influences and lifestyle [23]. However, to date no gene has been definitely identified as a major gene for OP.

The purpose of the present study is to evaluate the relation between BMD and genotypes of four SNPs in previously reported osteoporosis candidate-genes (*FDPS* rs2297480, *LRP5* rs3736228, *SOST* rs1234612, *VKORC1* rs9934438) in a cohort of postmenopausal Romanian women.

## Material and methods

### Study population

An analytical, prospective, transversal, observational, case-control study on 364 postmenopausal Romanian women was carried out between June 2016 and August 2017. All the women included in the study were recruited either from the inpatient clinic or during routine outpatient visit in the Clinical Rehabilitation Hospital in Cluj-Napoca, Romania.

The inclusion criteria was as follows: women, at least 45 years old, that had been menopausal for at least 2 years and had a recent diagnosis of osteoporosis or normal density result on BMD measurement using dual-energy X-ray absorbtiometry (DEXA). The women included in the study were divided into two groups: osteoporosis (n = 228) and healthy age-matched controls (n = 136). Patients with history of metabolic bone diseases (e.g. hyperparathyroidism, osteomalacia, Paget disease), malignancy, bone metastasis and those treated with drugs that influence bone metabolism (e.g. anti-osteoporotic drugs or vitamin K antagonists) were excluded from the study.

Clinical data was collected by interview and from the medical documents of each patient: age, body mass index, years since menopause, lumbar spine (L1-L4), femoral neck and total hip BMD. Furthermore, a 2 ml EDTA vial of peripheral blood was collected from each study participant for the genetic testing.

The study was approved by the Ethics Committee of the University of Medicine and Pharmacy “Iuliu Hațieganu” Cluj-Napoca (approval no. 248/09.06.2016). All participants were informed of the characteristics of the study and all gave signed informed consent regarding the genetic testing and clinical data collection prior to inclusion.

### SNP genotyping

Genomic DNA was obtained from peripheral blood withdrawn on EDTA, using commercially available kits (Quick gDNA MiniPrep kit, Zymo Research, USA; PureLink Genomic DNA Mini Kit, Invitrogen, Thermo Fisher, USA). We genotyped four SNPs (FDPS rs2297480, LRP5 rs3736228, SOST rs1234612, VKORC1 rs9934438) in all patients and controls using the real-time PCR technique. Standard, predesigned TaqMan SNP genotyping assays, containing all the primers and probes needed for genotyping, were purchased from Thermo Fisher (codes C___2737970_10, C__25752205_10, C___7566033_10, C__30204875_10). All the genotyping were performed according to manufacturer’s instructions. The reaction mix contained 10 μl of 2xTaqMan Genotyping Master Mix (Applied Biosystems, Thermo Fisher, USA), 0.5 μl of the corresponding 40xTaqMan SNP genotyping assay, approximately 25 ng of genomic DNA and free-nucleases water to the final volume of 20 μl. The same amplification program was used for all the genotyping, consisting in a pre-read stage of 30 seconds (s) at 60°C, hold stage of 10 minutes (min) at 95°C, followed by the PCR stage, consisting of 40 cycles, each comprising 15 s at 95°C and 1 min at 60°C, and a post-read stage of 30 s at 60°C. All the experiments were run on a QuantStudio 3 real-time PCR machine (Applied Biosystems, Thermo Fisher, USA).

### Statistical analysis

Statistical analysis was performed using MedCalc Statistical Software version 18.6 (MedCalc Software bvba, Ostend, Belgium; http://www.medcalc.org; 2018). Qualitative data were characterized by frequency and percent. Quantitative data were described by mean and standard deviation or median and 25–75 percentiles, depending on the normality of distribution at Kolmogorov Smirnov test. Differences between groups were tested using the chi-square or Mann-Whitney, whenever appropriate (t-test, Fischer). A p-value <0.05 was considered statistically significant. The size sample estimation was calculated by assessing the distribution of the TT genotype for FDPS rs2297480 SNP in a group of 23 patients (52.2%) with osteoporosis and 13 controls (38.5%). For a type I error of 0.05 and a type II error of 0.20, we calculated a number of 162 controls and 290 patients.

## Results

A total of 364 patients met the inclusion criteria and were included in the study to be genotyped, of which 228 had a diagnosis of OP and were compared to 136 age-matched healthy controls. The mean age in the osteoporosis group was 65.5 years (±7.39), and 63.45 (± 8.16) in the control group, respectively. The osteoporosis group had signficantly more years of amenorrhea than the control group, lower BMD values at all measured sites and a higher fracture risk (all p<0.05).

The main characteristics of the women included in the study are shown in Table 1.

**Table 1:** Clinical characteristics of women included in the study.

Variables	Osteoporosis *n* = 228	Controls *n* = 136	*p*-value
Age, mean ± SD [years]	65.5 ± 7.39	63.45 ± 8.16	**0.014**
BMI, mean ± SD [kg/m^2^]	27.05 ± 4.74	30.56 ± 5.40	**< 0.001**
Age at menopause, mean ± SD [years]	47.26 ± 4.84	48.35 ± 4.88	**0.045**
Time of amenorrhea, mean ± SD [years]	18.25 ± 8.36	15.08 ± 8.82	**0.001**
Previous fragility fracture (vertebral, hip, wrist, humerus), *n* (%)	132 (57.9)	0 (0)	-
Current smoking, *n* (%)	10 (4.4)	6 (4.4)	0.991
Alcohol consumption > 3 units/day, *n* (%)	3 (1.3)	0 (0)	-
Parent fractured hip, *n* (%)	13 (5.6)	8 (5.8)	0.943
Lumbar spine (L1-L4) BMD, mean ± SD [g/cm^2^]	0.851 ± 0.11	1.116 ± 0.15	**< 0.001**
Femoral neck BMD, mean ± SD [g/cm^2^]	0.751 ± 0.10	0.969 ± 0.21	**< 0.001**
Total hip BMD, mean ± SD [g/cm^2^]	0.791 ± 0.5	0.968 ± 0.21	**<0.001**
FRAX—10 year risk of major osteoporotic fracture, mean ± SD [%]	8.04 ± 4.68	4.34 ± 2.47	**0.005**
FRAX—10 year risk of hip fracture, mean ± SD [%]	2.76 ± 2.97	0.82 ± 1.47	**< 0.001**

The distribution of the *FDPS* rs2297480, *LRP5* rs3736228, *SOST* rs1234612 and *VKORC1* rs9934438 genotypes and allele frequencies in the study population (n = 364) are shown in Table 2.

**Table 2:** Allelic and genotypic frequencies of the SNPs in the study population (n = 364).

Gene	SNP	Genotypes (%)			Allele (%)	
*FPDS*	rs2297480	**TT**	**GT**	**GG**	**T**	**G**
		222 (60.99)	124 (34.07)	18 (4.95)	568 (78)	160 (22)
*LRP5*	rs3736228	**CC**	**CT**	**TT**	**C**	**T**
		257 (70.6)	103 (28.3)	4 (1.1)	617 (85)	111 (15)
*SOST*	rs1234612	**TT**	**CT**	**CC**	**T**	**C**
		183 (50.27)	142 (39.01)	39 (10.71)	508 (70)	220 (30)
*VKORC1*	rs9934438	**GG**	**AG**	**AA**	**G**	**A**
		126 (34.62)	156 (42.86)	82 (22.53)	408 (56)	320 (44)

Women carrying the TT genotype of *FDPS* rs2297480 have significantly lower BMD values in the lumbar spine and total hip than the heterozygous GT or homozygous GG (p = 0.006 and p = 0.03, respectively), but not in the femoral neck (p = 0.179) (Table 3).

**Table 3:** Bone mineral density values among genotypes (n = 364).

Gene (SNP)	Genotype	BMD L1-L4 g/cm^2^ (IQR)	BMD FEMORAL NECK g/cm^2^ (IQR)	BMD TOTAL HIP g/cm^2^ (IQR)
*FDPS* rs2297480	GG	0.910 (0.804; 1.023)	0.840 (0.699; 0.925)	0.865 (0.747; 1.030)
	GT	0.981 (0.852; 1.102)	0.803 (0.725; 0.885)	0.882 (0.779; 0.997)
	TT	0.865 (0.778; 1.091)	0.774 (0.705; 0.873)	0.833 (0.746; 0.939)
	p-value	**0.006**	0.179	**0.030**
*LRP5* rs3736228	TT	0.877 (0.669; 1.053)	0.782 (0.711; 0.861)	0.832 (0.756; 0.945)
	CT	0.943 (0.788; 1.099)	0.825 (0.714; 0.918)	0.895 (0,771; 1.019)
	CC	0.924 (0.836; 1.061)	0.715 (0.635; 0.747)	0.810 (0.644; 0.882)
	p-value	0.717	**0.028**	**0.014**
*SOST* rs1234612	CC	1.004 (0.897; 1.085)	0.792 (0.734; 0.891)	0.896 (0.742; 1.008)
	CT	0.941 (0.838; 1.076)	0.787 (0.702; 0.870)	0.850 (0.759; 0.950)
	TT	0.910 (0.812; 1.046)	0.799 (0.714; 0.886)	0.837 (0.757; 0.965)
	p-value	0.124	0.596	0.679
*VKORC1* rs9934438	AA	0.959 (0.848; 1.120)	0.803 (0.711; 0.931)	0.860 (0.770; 1.019)
	AG	0.910 (0.812; 1.055)	0.792 (0.702; 0.870)	0.832 (0.742; 0.948)
	GG	0.926 (0.823; 1.042)	0.794 (0.721; 0.873)	0.847 (0.771; 0.963)
	p-value	0.086	0.367	0.133

After dividing the patients into groups, analysis of the codominant model was performed. Codominant models hypothesize that the major allele homozygotes, the heterozygotes or the minor allele homozygotes are associated with the lowest, the intermediate, and the highest risk, respectively, or they are associated with the highest, the intermediate, and the lowest risk, respectively.

Results show that when analyzing the codominant model, the TT genotype of rs2297480 SNP continues to be significantly associated with OP risk (OR = 2.1; 95% CI = 1.4–3.3; p-value<0.05), while the GT genotype had the lowest risk (OR = 0.4; 95% CI = 0.3–0.7; p-value = 0.001). Furthermore, the presence of the T allele was significantly associated with OP in the group analysis (p = 0.005) (Table 4).

**Table 4:** Allelic and genotypic distribution between osteoporotic women and controls.

Gene	Polymorphism	Model/alleles	Genotypes, alleles	Osteoporosis (%) (n = 228)	Controls (%) n = 136	OR [95% CI]	p-value
FDPS	rs2297480	Codominant	GG	10 (4.4)	8 (5.9)	0.7 [0.2–1.9]	0.524
			GT	63 (27.6)	61 (44.9)	0.4 [0.3–0.7]	**0.001**
			TT	155 (68)	67 (49.3)	2.1 [1.4–3.3]	**<0.05**
		Major allele	T	373 (81.8)	195 (71.7)	-	**0.005**
		Minor allele	G	83 (18.2)	77 (28.3)	-	0.127
LRP5	rs3736228	Codominant	CC	169 (74.1)	88 (64.7)	1.5 [0.9–2.4]	**0.05**
			CT	56 (24.6)	47 (34.6)	0.6 [0.3–0.9] 0.6 [03.-0.9]	**0.041**
			TT	3 (1.3)	1 (0.7)	1.8 [0.1–17.4]	0.612
		Major allele	C	394 (86.4)	223 (82)	-	0.143
		Minor allele	T	62 (13.6)	49 (18)	-	0.527
SOST	rs1234612	Codominant	CC	19 (8.3)	20 (14.7)	0.5 [0.2–1]	0.057
			CT	88 (38.6)	54 (39.7)	0.9 [0.6–1.4]	0.834
			TT	121 (53.1)	62 (45.6)	1.3 [0.8–2]	0.167
		Major allele	T	330 (72.4)	178 (65.4)	-	0.101
		Minor allele	C	126 (27.6)	94 (34.6)	-	0.266
VKORC1	rs9934438	Codominant	AA	46 (20.2)	36 (26.5)	0.7 [0.4–1.1]	0.164
			AG	101 (44.3)	55 (40.4)	1.1 [0.7–1.8]	0.472
			GG	81 (35.5)	45 (33.1)	1.1 [0.7–1.7]	0.636
		Major allele	G	263 (57.7)	145 (53.3)	-	0.391
		Minor allele	A	193 (42.3)	127 (46.7)	-	0.438

As for *LRP5* rs3736228 SNP, the homozygous CC have significantly lower BMD values in the femoral neck and total hip than the CT or TT genotypes (p = 0.028 and p = 0.014), but not in the lumbar spine region (p = 0.717) (Table 3). In the codominant model of the groups analysis, this association continues to reach statistical significance in the OP group (OR = 1.5; 95% CI = 0.9–2.4; p = 0.05), while the heterozygous CT had the lowest risk (OR = 0.6; 95% CI = 0.3–0.9; p = 0.041). Furthermore, no association was found for the C allele and OP phenotype (p = 0.143) (Table 4).

No genotype or allele of *SOST* rs1234612 were found to be significant associated with OP or low BMD (all p>0.05) (Tables 3 and 4).

When analyzing BMD values between genotypes of *VKORC1* rs9934438, no statistical significance was found, except for the heterozygous AG which showed a slight trend of having lower BMD values in the lumbar spine (p = 0.086) (Table 3). The model and allelic analysis revealed no significant statistical associations between groups (all p>0.05) (Table 4).

## Discussion

Even though in the last two decades researchers have continuously searched for the role of genetic factors in the pathogenesis of bone loss, up to date, there is no conclusive etiologic information about OP in this area.

*FDPS* is one of the key-enzymes involved in the mevalonate pathway and it was identified as the main biochemical target of N-BPs [24]. Our research showed a significant association between the presence of the major allele T and osteoporosis (p = 0.005). Also, genotype TT of rs2297480 SNP had significantly lower BMD values in the lumbar spine and total hip (both p<0.05). These results are consistent with the findings of Levy at al. [17], who evaluated the same *FDPS* polymorphism and found a strong association between the presence of the major allele C and low BMD in elderly American women. Marini et al. [18], Olmos et al. [19] and Massart et al [25] evaluated the relation between genotypes of rs2297480 polymorphism and BMD in Caucasian women, but did not find an association with baseline BMD values. The genotype frequencies are similar to those reported in the American [17], Danish [18], Spanish [19] and Italian [25] population. The current findings are significant for Romanian postmenopausal women, as the T allele and the TT genotype are the most frequent (78% and 60.99%, respectively).

It is known that genes involved in the Wnt pathway are important players in skeletal homeostasis [26]. Polymorphisms in the *LRP5* gene have been previously linked to lumbar spine BMD in adults in multiple studies. Canto-Cetina et al. [27] found a significant association between rs3736228 polymorphism and variations in all BMD sites in Maya-Mestizo women. Interestingly, no significant association of the same SNP with BMD was found in the Slovenian [28], Mexican [29] or Chinese [30] population. In the present research, the CC and CT genotypes of *LRP5* rs3736228 were associated with OP (p = 0.05 and p = 0.041, respectively). Also, postmenopausal women carrying the CC genotype have significantly lower BMD values in the femoral neck and total hip (both p<0.05). Interestingly, in an Italian population, *LRP5* rs3736228 CC genotype tended to have higher BMD values than TT genotype in all BMD sites [25]. And Markatseli et al. [31] reported in a cohort of Greek peri- and postmenopausal women that the presence of CT/TT genotype is consistent with lower lumbar spine BMD. Ezura et al. [32] analyzed rs37362288 genotypes in relation to BMD in adult Japanese women. They found that the homozygous carrying the minor T allele had the lowest BMD scores, and that homozygous carrying the major C allele had the higher BMD. Interestingly, even though the genotypic distribution in the present study is similar to that reported in the Japanese [32], Italian [25] or Greek [31] women, our findings suggest that osteoporosis-risk genotype could be the homozygous CC in postmenopausal Romanian women.

There has been evidence that other Wnt genes, like *SOST*, influence BMD in the general population [33,34]. Valero et al. [35] found that several polymorphisms in the 5’ region of the *SOST* gene are associated with BMD in postmenopausal Spanish women. The CTT haplotype at loci rs1234612 was significantly associated with lumbar spine BMD, but not in the femoral neck area. The present study found no association between genotypes and BMD values (all p>0.05). Zhang et al. [36] found that the rs1234612 and the *SOST* haplotype GGTGGATC were associated with adjusted total hip BMD in a large sample of postmenopausal Chinese women. On the contrast, He and al. [37] and Balemans et al. [38] did not find any association between *SOST* gene polymorphisms and BMD in postmenopausal and perimenopausal women, respectively. Velasquez-Cruz et al. [39] concluded that *SOST* polymorphisms contribute to total hip and femoral neck BMD in postmenopausal Mexican-mestizo women. Zhou et al. [36] evaluated several *SOST* polymorphisms regarding treatment response to N-BPs therapy. They found a strong correlation between subjects with homozygous common alleles of rs1234612 and rs851054 and baseline BMD in the lumbar spine. In our study, genotype frequencies of *SOST* rs1234612 were not different than previously reported in the Asian [36,40] population.

There has been evidence that vitamin K plays an important role in maintaining bone strength by acting as a cofactor of the gamma-carboxylase enzyme which activates Vitamin K-dependent proteins in bone, like osteocalcin [41,42]. The *VKORC1* gene is highly polymorphic and mutations in the *VKORC1* gene may modify the gamma-carboxylation of osteocalcin and may influence BMD [20,21]. The effects of *VKORC1* gene on bone have been investigated in several studies. Crawford et al. [43] showed an association of several SNPs in the *VKORC1* gene with BMD in African-Americans. Also, Holzer et al. [44] made a significant association between 3673 G>A genotype and BMD, showing that the homozygous AA had lower BMD values than AG or GG. In the current research, the heterozygous AG tended to have lower BMD values in the lumbar spine area, but it did not reach statistical significance (p = 0.086). Interestingly, Fodor et al. [20] found that the TT genotype of *VKORC1* 1173C>T was the most frequent in another cohort of postmenopausal Romanian women with OP or osteopenia. Also, a recent study conducted on a Turkish population showed no association between a polymorphism in the *VKORC1* gene and BMD [21]. In our study, the genotype frequencies of the rs9934438 polymorphism were similar to those previously reported in the Romanian [20,22] population. However, relevant studies demonstrating the relationship of this gene to bone biology are still lacking.

This study has several limitations. First, it included only white women and it is unclear if the results can be extrapolated to women of different ethnicity. Secondly, the cohort size was modest, as genetic investigation requires higher number of subjects to achieve sufficient statistical power. And thirdly, the study design was clinic and volunteer based, not population based, therefore potential biases remain.

To our knowledge, this is the first study to detect a positive or negative association between four SNPs in different genes with BMD in a Romanian cohort of postmenopausal women with OP. To date, only one of the SNPs included in this study has been evaluated before in a Romanian population regarding its relation with BMD (*VKORC1* rs9934438) [23]. Also, it is important to mention that the Romanian population is relatively understudied with regards to OP genetics, and the results from this study are potentially of interest for future meta-analyses. Because of the polygenic nature of OP, few genes have major impact on bone metabolism or homeostasis and multiple genes have minor effects, making the classical gene-disease association approach limited, as it mostly lead to inconclusive or controversial results. Regardless of this, data obtained from the current study could be important to Romanian postmenopausal women, as the burden of OP and osteoporosis-related fractures is increasing in Romania, as in other European countries [45].

## Conclusions

Our study showed a strong association between osteoporosis and TT genotype of *FDPS* rs2297480, and with CC genotype of *LRP5* rs3736228. No statistical significance was found between genotypes of *SOST* rs1234612 and *VKORC1* rs9934438 with BMD in postmenopausal Romanian women with osteoporosis.

## Supporting information

S1 File Table with genotyping data and bone mineral density. (PDF)

## References

[1] Cummings SR, Melton LJ. Epidemiology and outcomes of osteoporotic fractures. Lancet 2002;359:1761–1767. 10.1016/S0140-6736(02)08657-9 12049882

[2] Rivadeneira F, Mäkitie O. Osteoporosis and bone mass disorders: from gene pathways to treatments. Trends in Endocrinology & Metabolism. 2016 5 1;27(5):262–81. 27079517 10.1016/j.tem.2016.03.006

[3] Ralston SH, Uitterlinden AG. Genetics of osteoporosis. Endocr Rev 2010;31:629–662. 10.1210/er.2009-0044 20431112

[4] Zofkova I, Nemcikova P, Kuklik M. Polymorphisms associated with low bone mass and high risk of atraumatic fracture. Physiological research 2015;64:621–631. 25804099 10.33549/physiolres.932973

[5] Rivadeneira F, Mäkitie O. Osteoporosis and bone mass disorders: from gene pathways to treatments. Trends in Endocrinology & Metabolism. 2016;27(5):262–81. 27079517 10.1016/j.tem.2016.03.006

[6] Angers S, Moon RT. Proximal events in Wnt signal transduction. Nature reviews Molecular cell biology. 2009;10(7):468 10.1038/nrm2717 19536106

[7] Richards JB, Rivadeneira F, Inouye M, Pastinen TM, Soranzo N, Wilson SG, et al Bone mineral density, osteoporosis, and osteoporotic fractures: a genome-wide association study. The Lancet. 2008;371(9623):1505–12. 10.1016/S0140-6736(08)60599-1 PMC2679414 18455228

[8] Levasseur R, Lacombe D, de Vernejoul MC. LRP5 mutations in osteoporosis-pseudoglioma syndrome and high-bone-mass disorders. Joint Bone Spine. 2005;72(3):207–14. 10.1016/j.jbspin.2004.10.008 15850991

[9] Golchin MM, Heidari L, Ghaderian SM, Akhavan-Niaki H. Osteoporosis: a silent disease with complex genetic contribution. Journal of Genetics and Genomics. 2016;43(2):49–61. 10.1016/j.jgg.2015.12.001 26924688

[10] Winkler DG, Sutherland MK, Geoghegan JC, Yu C, Hayes T, Skonier JE, et al Osteocyte control of bone formation via sclerostin, a novel BMP antagonist. EMBO J. 2003;22:6267–76. 10.1093/emboj/cdg599 14633986 PMC291840

[11] Loots GG, Kneissel M, Keller H, Baptist M, Chang J, Collette NM, et al Genomic deletion of a long-range bone enhancer misregulates sclerostin in Van Buchem disease. Genome Res. 2005;15:928–35 10.1101/gr.3437105 15965026 PMC1172036

[12] Collette NM, Genetos DC, Economides AN, Xie L, Shahnazari M, Yao W, et al Targeted deletion of Sost distal enhancer increases bone formation and bone mass. Proc Natl Acad Sci USA. 2012;109:14092–7 10.1073/pnas.1207188109 22886088 PMC3435175

[13] Marozik P, Alekna V, Rudenko E, Tamulaitiene M, Rudenka A, Mastaviciute A, et al Bone metabolism genes variation and response to bisphosphonate treatment in women with postmenopausal osteoporosis. PloS one. 2019;14(8). 10.1371/journal.pone.0221511 PMC6705789 31437227

[14] Choi HJ, Choi JY, Cho SW, Kang D, Han KO, Kim SW, et al Genetic polymorphism of geranylgeranyl diphosphate synthase (GGSP1) predicts bone density response to bisphosphonate therapy in Korean women. Yonsei medical journal. 2010;51(2):231–8. 10.3349/ymj.2010.51.2.231 20191015 PMC2824869

[15] Wang C, Zheng H, He JW, Zhang H, Yue H, Hu WW, et al Genetic polymorphisms in the mevalonate pathway affect the therapeutic response to alendronate treatment in postmenopausal Chinese women with low bone mineral density. The pharmacogenomics journal. 2015;15(2):158 10.1038/tpj.2014.52 25223561

[16] Han LW, Ma DD, Xu XJ, Lü F, Liu Y, Xia WB, et al Association Between Geranylgeranyl Pyrophosphate Synthase Gene Polymorphisms and Bone Phenotypes and Response to Alendronate Treatment in Chinese Osteoporotic Women. Chinese Medical Sciences Journal. 2016;31(1):8–16. 10.1016/s1001-9294(16)30016-5 28031082

[17] Levy ME, Parker RA, Ferrell RE, Zmuda JM, Greenspan SL. Farnesyl diphosphate synthase: a novel genotype association with bone mineral density in elderly women. Maturitas 2007;57(3):247–252. 10.1016/j.maturitas.2007.01.005 17368768 PMC1951796

[18] Marini F, Falchetti A, Silvestri S, Bagger Y, Luzi E, Tanini A, et al Modulatory effect of farnesyl pyrophosphate synthase (FDPS) rs2297480 polymorphism on the response to long-term amino-bisphosphonate treatment in postmenopausal osteoporosis. Current Medical research and opinion. 2008;24(9):2609–15. 10.1185/03007990802352894 18687167

[19] Olmos JM, Zarrabeitia MT, Hernandez JL, Sanudo C, Gonzalez-Macias J, Riancho JA. Common allelic variants of the farnesyl diphosphate synthase gene influence the response of osteoporotic women to bisphosphonates. The pharmacogenomics journal 2012;12(3):227 10.1038/tpj.2010.88 21151198

[20] Fodor D, Bondor C, Albu A, Popp R, Pop IV, Poanta L. Relationship between VKORC1 single nucleotide polymorphism 1173C> T, bone mineral density & carotid intima-media thickness. The Indian journal of medical research 2013;137(4):734 23703341 PMC3724254

[21] Kutluturk F, Inanir A, Rustemoglu A, Kaya SU, Demir AK, Dursun G, et al Association between Vitamin K Epoxide Reductase (VKORC1)-1639G> A Polymorphism and Osteoporosis in Postmenopausal Women. Endocrine, Metabolic & Immune Disorders-Drug Targets (Formerly Current Drug Targets-Immune, Endocrine & Metabolic Disorders). 2018;18(3):281–6. 10.2174/1871530318666180212100119 29437025

[22] Pop TR, Vesa ŞC, Trifa AP, Crişan S, Buzoianu AD. An acenocoumarol dose algorithm based on a South-Eastern European population. European journal of clinical pharmacology 2013;69(11):1901–1907. 10.1007/s00228-013-1551-3 23774941

[23] Marini F, Masi L, Marcucci G, Cianferotti L, Brandi ML. Genetics of Osteoporosis In Multidisciplinary Approach to Osteoporosis. Cham: Springer;2018 p. 25–44

[24] van Beek E, Pieterman E, Cohen L, Löwik C, Papapoulos S. Farnesyl pyrophosphate synthase is the molecular target of nitrogen-containing bisphosphonates. Biochemical and biophysical research communications 1999;264(1):108–111. 10.1006/bbrc.1999.1499 10527849

[25] Massart F, Marini F, Bianchi G, Minisola S, Luisetto G, Pirazzoli A, et al Genetic predictors of skeletal outcomes in healthy fertile women: The Bonturno Study. Joint Bone Spine. 2013;80(4):414–9. 10.1016/j.jbspin.2012.10.020 23238007

[26] Harada SI, Rodan GA. Control of osteoblast function and regulation of bone mass. Nature 2003;423(6937):349 10.1038/nature01660 12748654

[27] Canto‐Cetina T, Polanco Reyes L, González Herrera L, Rojano‐Mejía D, Coral‐Vázquez RM, Coronel A, et al Polymorphism of LRP5, but not of TNFRSF11B, is associated with a decrease in bone mineral density in postmenopausal maya‐mestizo women. American Journal of Human Biology. 2013;25(6):713–8. 10.1002/ajhb.22464 24130145

[28] Mencej-Bedrač S, Preželj J, Kocjan T, Komadina R, Marc J. Analysis of association of LRP5, LRP6, SOST, DKK1, and CTNNB1 genes with bone mineral density in a Slovenian population. Calcified tissue international 2009;85(6):501–506. 10.1007/s00223-009-9306-y 19898734

[29] Falcón-Ramírez E, Casas-Avila L, Cerda-Flores RM, Castro-Hernández C, Rubio-Lightbourn J, Velázquez-Cruz R, et al Association of LRP5 haplotypes with osteoporosis in Mexican women. Molecular biology reports. 2013;40(3):2705–10. 10.1007/s11033-012-2357-6 23242660

[30] Zhou PR, Liu HJ, Liao EY, Zhang ZL, Chen DC, Liu J, et al LRP5 polymorphisms and response to alendronate treatment in Chinese postmenopausal women with osteoporosis. Pharmacogenomics. 2014;15(6):821–31. 10.2217/pgs.14.12 24897288

[31] Markatseli AE, Hatzi E, Bouba I, Georgiou I, Challa A, Tigas S, et al Association of the A1330V and V667M polymorphisms of LRP5 with bone mineral density in Greek peri-and postmenopausal women. Maturitas. 2011;70(2):188–93. 10.1016/j.maturitas.2011.07.016 21840657

[32] Ezura Y, Nakajima T, Urano T, Sudo Y, Kajita M, Yoshida H, et al Association of a single-nucleotide variation (A1330V) in the low-density lipoprotein receptor-related protein 5 gene (LRP5) with bone mineral density in adult Japanese women. Bone. 2007;40(4):997–1005. 10.1016/j.bone.2005.06.025 17306638

[33] Martínez-Gil N, Roca-Ayats N, Monistrol-Mula A, García-Giralt N, Díez-Pérez A, Nogués X, et al Common and rare variants of WNT16, DKK1 and SOST and their relationship with bone mineral density. Scientific reports. 2018;8(1):10951 10.1038/s41598-018-29242-8 30026596 PMC6053384

[34] Uitterlinden AG, Arp PP, Paeper BW, Charmley P, Proll S, Rivadeneira F, et al Polymorphisms in the sclerosteosis/van Buchem disease gene (SOST) region are associated with bone-mineral density in elderly whites. The American Journal of Human Genetics. 2004;75(6):1032–45. 10.1086/426458 15514891 PMC1182139

[35] Valero C, Zarrabeitia MT, Hernández JL, Pineda B, Cano A, García-Pérez MA, et al Relationship of sclerostin and secreted frizzled protein polymorphisms with bone mineral density: an association study with replication in postmenopausal women. Menopause. 2011;18(7):802–7. 10.1097/gme.0b013e3182091664 21441835

[36] Zhou PR, Xu XJ, Zhang ZL, Liao EY, Chen DC, Liu J, et al SOST polymorphisms and response to alendronate treatment in postmenopausal Chinese women with osteoporosis. Pharmacogenomics. 2015;16(10):1077–88. 10.2217/pgs.15.76 26250343

[37] He J, Zhang H, Wang C, Zhang Z, Yue H, Hu W, et al Associations of serum sclerostin and polymorphisms in the SOST gene with bone mineral density and markers of bone metabolism in postmenopausal Chinese women. The Journal of Clinical Endocrinology & Metabolism. 2014;99(4):E665–73. 24423318 10.1210/jc.2013-2086

[38] Balemans W, Foernzler D, Parsons C, Ebeling M, Thompson A, Reid DM, et al Lack of association between the SOST gene and bone mineral density in perimenopausal women: analysis of five polymorphisms. Bone. 2002;31(4):515–9. 10.1016/s8756-3282(02)00844-x 12398949

[39] Velázquez-Cruz R, Jiménez-Ortega RF, Parra-Torres AY, Castillejos-López M, Patiño N, Quiterio M, et al Analysis of association of MEF2C, SOST and JAG1 genes with bone mineral density in Mexican-Mestizo postmenopausal women. BMC musculoskeletal disorders. 2014;15(1):400. 25430630 10.1186/1471-2474-15-400 PMC4258010

[40] Zhang H, He JW, Wang C, Zhang Z, Yue H, Hu WW, et al Associations of polymorphisms in the SOST gene and bone mineral density in postmenopausal Chinese Women. Osteoporosis International. 2014;25(12):2797–803. 10.1007/s00198-014-2832-0 25103216

[41] Palermo A, Tuccinardi D, D’Onofrio L, Watanabe M, Maggi D, Maurizi AR, et al Vitamin K and osteoporosis: Myth or reality?. Metabolism. 2017;70:57–71. 10.1016/j.metabol.2017.01.032 28403946

[42] Atkins GJ, Welldon KJ, Wijenayaka AR, Bonewald LF, Findlay DM. Vitamin K promotes mineralization, osteoblast-to-osteocyte transition, and an anticatabolic phenotype by γ-carboxylation-dependent and-independent mechanisms. American Journal of Physiology-Cell Physiology 2009;297(6):C1358–1367. 10.1152/ajpcell.00216.2009 19675304

[43] Crawford DC, Brown-Gentry K, Rieder MJ. VKORC1 common variation and bone mineral density in the Third National Health and Nutrition Examination Survey. PLoS One 2010;5(12):e15088 10.1371/journal.pone.0015088 21179439 PMC3001474

[44] Holzer G, Grasse AV, Zehetmayer S, Bencur P, Bieglmayer C, Mannhalter C. Vitamin K epoxide reductase (VKORC1) gene mutations in osteoporosis: A pilot study. Translational Research 2010;156(1):37–44. 10.1016/j.trsl.2010.05.005 20621035

[45] Hernlund E, Svedbom A, Ivergård M, Compston J, Cooper C, Stenmark J, et al Osteoporosis in the European Union: medical management, epidemiology and economic burden. Archives of osteoporosis. 2013;8(1–2):136. 24113837 10.1007/s11657-013-0136-1 PMC3880487

