# Supplementary material for: Polymorphisms of FDPS, LRP5, SOST and VKORC1 genes and their relation with osteoporosis in postmenopausal Romanian women
Source: PLoS One. 2019 Nov 27;14(11):e0225776. doi: 10.1371/journal.pone.0225776 (PMC6880991; doi:10.1371/journal.pone.0225776)
Supplement: S1 File — (PDF) [file pone.0225776.s001.pdf]

ID	BMD L1-L4	BMD FN	BMD TH	rs2297480 - FDPS	rs3736228 - LRP5	rs 1234612 - SOST	rs 9934438 - VKORC1
1	1.051	1.338	1.333	12	12	11	22
2	0.848	1.05	0.815	22	11	12	11
3	1.173	1.055	0.997	12	11	22	12
4	0.822	0.631	0.804	22	11	22	11
5	0.812	0.813	0.883	12	11	12	11
6	0.973	0.983	1.064	22	12	12	22
7	1.119	0.796	0.931	11	11	12	12
8	1.153	0.792	0.812	22	11	12	12
9	1.034	0.983	1.059	22	12	22	22
10	1.091	0.693	0.744	11	11	12	12
11	1.199	0.929	1.071	11	11	12	11
12	0.762	0.886	0.765	22	12	22	12
13	1.465	0.971	1.106	12	12	22	11
14	0.959	0.664	0.719	22	11	22	11
15	0.800	0.804	0.834	12	11	12	22
16	0.384	0.784	0.813	22	11	11	11
17	1.175	1.055	1.122	12	11	22	11
18	1.085	0.813	0.916	22	11	11	12
19	1.123	0.842	0.997	22	12	11	22
20	0.961	0.727	0.804	22	11	22	22
21	0.910	0.892	0.921	22	12	11	12
22	1.186	0.779	0.815	22	11	11	12
23	0.813	0.793	0.765	22	11	22	12
24	0.971	0.669	0.813	22	11	22	22

25	0.894	0.808	0.883	22	11	22	11
26	1.077	0.96	1.067	12	11	12	11
27	0.703	0.663	0.718	22	12	22	12
28	1.007	1.004	1.078	22	12	12	12
29	1.040	0.812	0.96	22	11	22	12
30	1.171	0.971	1.019	12	11	22	22
31	0.999	0.754	0.904	22	11	22	22
32	1.513	1.14	1.178	22	11	12	11
33	1.193	0.78	0.98	12	11	12	22
34	1.150	0.922	0.984	12	11	22	11
35	1.022	0.573	0.638	22	11	22	12
36	0.894	0.745	0.866	22	12	22	22
37	0.937	0.784	0.631	12	11	11	11
38	0.970	0.793	0.806	22	11	11	22
39	0.790	0.825	0.79	12	12	22	12
40	0.991	0.819	0.937	12	12	22	12
41	1.148	0.664	0.791	22	11	12	11
42	1.011	0.877	0.915	22	12	12	12
43	1.150	0.886	1.041	22	11	22	12
44	0.937	0.925	0.973	22	12	22	22
45	1.050	0.793	0.846	12	11	12	12
46	1.023	0.966	1.053	22	11	22	12
47	1.006	0.653	0.896	12	12	11	11
48	1.059	0.891	1.001	11	11	11	22
49	1.003	0.921	0.976	22	12	22	11

50	0.958	0.896	1.042	22	11	12	22
51	1.153	0.792	0.812	22	11	11	12
52	0.978	0.734	0.895	22	11	12	12
53	1.190	0.74	0.854	12	12	22	22
54	0.737	0.736	0.715	22	12	22	22
55	1.414	0.921	1.007	22	11	22	22
56	1.126	0.873	1.097	12	12	12	11
57	1.018	0.873	0.965	22	11	22	12
58	1.574	1.147	1.297	12	12	22	12
59	0.916	0.844	0.972	12	11	22	11
60	0.980	0.958	1.024	22	11	11	12
61	0.826	0.747	0.806	12	12	22	22
62	1.068	0.886	1.028	12	12	11	12
63	0.965	0.747	0.806	12	11	22	11
64	1.300	0.867	0.693	22	12	12	12
65	1.177	0.843	0.823	12	11	12	11
66	0.966	0.853	0.912	12	11	12	12
67	1.061	0.989	1.028	12	11	12	12
68	0.949	0.849	0.892	12	12	12	12
69	1.303	0.931	1.052	12	12	22	12
70	0.965	0.747	0.806	22	11	22	12
71	1.297	1.117	1.062	12	12	12	12
72	1.171	0.971	1.019	22	12	12	11
73	1.141	0.663	0.876	12	11	12	22
74	0.701	0.676	0.78	12	11	12	12

75	1.085	1.195	1.168	22	11	12	11
76	1.154	0.943	0.998	22	12	22	22
77	0.800	0.804	0.834	22	11	22	11
78	0.924	0.713	0.826	22	11	22	11
79	0.877	0.643	0.792	12	11	12	12
80	0.959	0.664	0.719	22	11	22	11
81	1.447	0.743	0.8	22	12	12	11
82	1.293	1.314	1.407	22	12	22	12
83	1.029	0.895	1.056	22	11	22	11
84	0.844	0.748	0.826	12	11	22	12
85	0.923	0.859	1.068	22	12	22	22
86	0.990	0.842	0.915	22	11	22	22
87	1.204	0.852	0.969	12	11	22	22
88	0.872	0.981	1.06	22	11	22	12
89	0.956	0.708	0.7	22	11	12	12
90	0.693	0.67	0.704	22	11	22	12
91	0.886	0.879	0.892	22	12	12	11
92	1.062	0.965	1.174	22	11	11	22
93	1.096	0.69	0.782	22	11	22	22
94	0.861	0.624	0.684	12	11	12	12
95	1.409	1.025	1.078	12	12	12	12
96	1.119	0.796	0.931	12	12	12	11
97	1.093	0.781	0.78	12	11	22	11
98	0.944	0.744	0.852	22	11	11	11
99	1.059	0.659	0.762	12	11	22	12

100	0.965	0.904	0.979	12	11	12	22
101	1.069	0.705	0.848	12	11	22	12
102	1.030	0.756	0.832	22	11	12	22
103	1.115	0.813	0.955	12	11	22	22
104	0.897	0.791	0.795	12	11	11	22
105	1.099	0.928	1.063	22	11	11	11
106	1.243	0.761	0.948	22	11	22	22
107	0.966	0.664	0.743	22	11	22	12
108	0.890	0.721	0.877	22	11	12	22
109	1.051	0.765	0.851	11	12	12	12
110	0.909	0.665	0.855	22	11	12	12
111	1.139	0.843	0.836	22	12	12	11
112	0.969	0.81	0.884	11	11	12	12
113	1.216	0.933	1.116	12	11	22	11
114	0.979	0.767	0.816	22	11	12	11
115	0.817	0.807	0.889	22	11	12	11
116	1.188	0.98	1.133	22	11	12	11
117	0.687	0.654	0.679	12	11	12	12
118	0.783	0.714	0.886	22	12	12	12
119	1.103	0.805	0.928	12	12	12	12
120	0.904	1.011	1.024	22	11	22	12
121	0.923	0.777	0.85	12	11	12	11
122	1.011	0.641	0.732	22	11	12	11
123	0.883	0.479	0.515	22	11	22	12
124	0.707	0.637	0.66	22	11	22	12

125	1.096	1.175	1.26	12	11	22	22
126	0.814	0.634	0.744	12	11	22	12
127	0.876	0.773	0.751	22	11	22	11
128	0.976	0.843	0.95	22	12	12	12
129	1.100	0.862	0.918	22	11	12	22
130	0.749	0.702	0.706	11	12	12	22
131	1.302	0.973	1.008	22	12	11	11
132	0.993	0.75	0.884	12	22	22	12
133	1.078	0.841	0.825	22	11	22	12
134	1.404	0.745	0.887	22	11	12	12
135	1.074	0.944	1.029	22	12	11	11
136	0.914	0.754	0.697	12	11	12	22
137	1.068	0.855	0.902	22	11	11	12
138	0.933	0.816	0.922	22	11	22	22
139	0.888	0.67	0.677	22	12	12	22
140	0.691	0.671	0.735	22	12	12	11
141	1.099	0.898	0.843	22	11	22	12
142	1.396	1.099	1.114	22	11	22	22
143	0.827	0.682	0.77	12	12	22	22
144	1.067	0.896	1.02	22	12	12	22
145	0.747	0.605	0.743	12	12	12	22
146	0.970	0.839	0.868	22	12	12	22
147	1.404	0.745	1.003	22	12	12	12
148	1.087	1.067	1.196	12	11	22	11
149	0.691	0.671	0.735	22	12	11	22

150	0.640	0.82	0.807	22	11	22	22
151	1.582	1.018	1.046	22	11	12	11
152	1.074	0.616	0.742	22	22	11	12
153	0.940	0.761	0.835	22	11	12	22
154	0.821	0.782	0.837	12	11	12	22
155	1.053	0.773	0.854	22	11	12	12
156	1.397	1.291	1.396	12	11	12	12
157	1.040	0.723	0.764	12	11	12	11
158	1.109	0.771	0.959	12	11	11	22
159	0.951	0.701	0.756	22	11	22	11
160	1.026	0.799	0.89	22	11	22	12
161	0.758	0.748	0.735	22	11	11	12
162	0.786	0.81	0.851	22	11	12	11
163	0.690	0.645	0.704	22	12	22	22
164	1.080	0.824	0.837	12	11	22	12
165	0.902	0.863	0.962	22	12	22	22
166	0.881	0.819	0.817	12	12	22	22
167	0.954	0.821	0.914	22	12	22	22
168	1.102	0.619	0.793	22	11	12	12
169	1.094	1.095	1.221	11	11	22	22
170	1.046	0.916	0.978	22	12	22	12
171	0.764	0.931	0.934	22	12	22	11
172	0.989	0.958	0.981	12	11	22	22
173	0.954	0.821	0.914	12	11	12	12
174	1.032	0.876	-0.955	22	11	22	12

175	1.282	0.962	1.036	12	11	22	11
176	1.091	0.85	0.93	22	11	11	11
177	0.690	0.645	0.704	22	11	22	11
178	1.071	0.842	0.834	12	11	22	22
179	0.956	0.708	0.7	22	11	12	11
180	0.968	0.967	1.097	12	11	22	12
181	1.057	0.723	0.822	12	11	22	12
182	0.701	0.676	0.78	22	11	22	22
183	0.912	0.835	0.99	22	11	22	12
184	1.105	0.839	0.929	22	11	22	12
185	0.760	0.848	0.901	12	11	12	22
186	1.171	0.971	1.019	12	11	22	11
187	0.992	0.842	0.882	12	11	22	22
188	0.989	0.871	0.878	22	11	12	22
189	1.077	1.265	1.295	22	11	12	22
190	0.952	0.653	0.896	12	12	11	11
191	1.091	0.748	0.757	12	11	22	12
192	0.848	1.05	0.815	22	11	22	22
193	1.153	0.792	0.812	12	11	22	22
194	0.828	0.776	0.72	11	11	22	12
195	0.822	0.631	0.804	22	11	11	12
196	1.247	1.069	1.192	22	11	12	12
197	0.855	0.845	0.862	22	11	12	11
198	1.238	1.061	1.171	12	11	22	22
199	1.375	1.116	1.227	22	11	22	12

200	0.895	0.927	0.952	22	11	12	22
201	0.915	0.81	0.896	22	12	12	22
202	1.146	0.948	1.035	22	12	12	22
203	1.280	0.901	0.958	22	12	22	22
204	1.324	0.853	1.055	12	12	12	22
205	1.070	0.649	0.747	12	11	12	11
206	0.863	0.781	0.87	12	11	22	11
207	0.963	0.731	0.894	22	11	22	11
208	0.872	0.888	1.005	22	11	22	12
209	0.872	0.877	0.89	22	12	22	22
210	0.857	0.76	0.848	22	11	22	12
211	1.152	0.862	0.932	12	12	22	11
212	1.016	0.834	0.824	22	11	11	22
213	0.741	0.82	0.802	12	12	12	12
214	0.758	0.759	0.748	22	11	22	22
215	1.004	0.803	0.919	12	12	11	11
216	1.176	1.112	1.141	22	12	22	22
217	0.909	0.534	0.584	22	11	12	12
218	1.097	0.764	0.854	22	12	22	12
219	0.958	0.726	0.85	22	11	22	12
220	0.870	0.807	0.892	22	11	12	12
221	1.081	0.749	0.843	22	11	22	22
222	0.909	0.534	0.584	22	11	12	11
223	0.978	0.734	0.895	22	12	11	12
224	0.967	0.858	0.827	22	11	22	12

225	171.575	0.668	0.737	22	11	12	12
226	1.008	0.986	1.007	12	12	12	12
227	0.827	0.757	0.916	12	11	22	22
228	0.936	0.817	0.772	22	12	22	22
229	0.853	0.873	0.67	22	11	22	22
230	0.771	0.691	0.88	22	12	22	22
231	0.805	0.752	0.779	12	11	22	12
232	1.123	0.842	0.997	12	11	22	12
233	0.924	0.713	0.826	22	11	22	12
234	1.077	0.96	1.067	12	11	12	11
235	0.946	0.702	0.834	12	11	12	12
236	0.870	0.709	0.772	12	11	22	12
237	0.855	0.75	0.93	12	11	12	12
238	0.851	0.851	0.689	22	11	22	12
239	1.086	0.627	0.726	22	11	12	12
240	0.900	0.823	0.73	12	11	22	22
241	0.897	0.815	0.813	12	11	12	12
242	0.943	0.692	0.883	12	12	12	22
243	0.601	0.642	0.655	22	11	22	22
244	0.930	0.945	0.944	22	11	22	12
245	0.652	0.623	0.689	22	12	12	12
246	0.969	0.661	0.68	22	12	12	22
247	0.738	0.779	0.638	12	11	22	12
248	0.804	0.659	0.643	22	12	22	11
249	0.741	0.948	0.833	22	11	12	11

250	0.868	0.688	0.698	22	11	22	12
251	0.975	0.721	0.802	12	11	12	22
252	0.895	0.744	0.823	22	11	22	22
253	0.671	1.12	0.837	22	12	22	12
254	0.737	0.863	1.063	22	12	11	12
255	0.650	0.661	0.653	12	11	11	12
256	0.735	0.496	0.504	12	11	12	22
257	0.994	0.738	0.83	22	12	22	12
258	0.795	0.727	0.789	22	12	12	11
259	1.016	0.701	0.746	12	11	12	22
260	0.959	0.664	0.719	12	11	12	22
261	0.769	0.717	0.76	22	11	12	22
262	0.764	0.614	0.735	22	11	22	22
263	0.807	0.545	0.63	22	11	12	12
264	0.812	0.669	0.817	22	11	12	22
265	0.862	0.805	0.881	12	11	22	22
266	1.192	0.67	0.779	12	11	22	22
267	0.834	0.759	0.636	12	11	12	12
268	0.624	0.656	0.672	12	12	22	12
269	0.762	0.739	0.879	22	22	12	11
270	0.703	0.7	0.757	22	12	12	11
271	0.761	0.705	0.76	22	12	22	12
272	0.805	0.925	0.827	11	11	22	12
273	0.864	0.833	0.87	12	11	22	22
274	0.951	0.701	0.756	22	11	12	11

275	0.894	0.615	0.777	22	12	12	22
276	0.841	0.647	0.647	22	11	12	12
277	0.888	0.793	0.735	22	11	22	12
278	0.712	0.692	0.791	11	11	22	12
279	0.814	0.839	1.039	12	11	22	12
280	1.513	1.14	1.178	12	12	11	22
281	0.691	0.622	0.661	22	11	22	12
282	1.039	0.742	0.864	22	11	22	22
283	0.879	0.795	0.766	22	11	12	12
284	0.915	0.797	0.869	22	11	12	12
285	0.796	0.802	0.836	22	11	12	22
286	1.397	1.291	1.396	11	11	11	11
287	0.671	0.608	0.623	11	12	22	12
288	0.639	0.692	0.612	22	22	22	12
289	0.850	0.86	0.649	22	11	22	12
290	0.924	0.713	0.826	22	11	12	22
291	0.717	0.651	0.608	12	11	12	12
292	0.932	0.839	0.859	22	11	12	11
293	0.734	0.666	0.7	22	11	12	22
294	0.724	0.739	0.784	22	11	22	22
295	0.911	0.762	0.599	22	11	11	12
296	0.766	0.612	0.654	22	11	12	22
297	0.944	0.744	0.852	22	11	22	22
298	0.940	0.508	0.562	12	12	22	12
299	0.853	0.753	0.966	12	11	22	12

300	0.787	0.769	0.747	22	11	22	11
301	0.855	0.706	0.797	22	11	12	12
302	0.895	1	1.049	22	12	22	12
303	0.795	0.684	0.745	22	11	22	12
304	0.785	0.736	0.792	22	11	22	12
305	0.883	0.813	0.737	22	11	12	12
306	0.872	0.769	0.851	22	11	12	12
307	0.734	0.714	0.629	22	12	22	22
308	0.835	0.683	0.773	12	11	12	11
309	0.735	0.744	0.603	22	11	12	11
310	0.710	0.776	0.729	22	12	11	22
311	0.910	0.667	0.641	22	11	22	12
312	0.824	0.801	0.813	12	11	22	22
313	0.872	0.835	0.942	22	11	22	12
314	0.856	0.903	0.88	11	12	22	22
315	0.769	0.84	0.956	22	12	22	22
316	0.656	0.832	0.795	22	11	12	12
317	0.797	0.884	892	12	11	12	22
318	0.993	0.75	0.884	12	11	12	12
319	0.788	0.87	1.028	11	12	22	12
320	0.848	0.878	0.946	22	11	22	11
321	0.711	0.773	0.723	12	11	12	12
322	0.905	0.87	0.958	12	11	12	12
323	0.883	0.722	0.826	22	11	22	22
324	0.899	0.95	1.001	22	11	12	12

325	1.059	0.891	1.001	12	11	22	12
326	0.747	0.881	0.756	12	11	22	12
327	0.597	0.537	0.763	11	12	22	12
328	0.866	0.83	0.938	22	11	12	12
329	0.825	0.873	0.966	22	11	22	12
330	0.842	0.916	0.978	22	11	22	22
331	0.734	0.714	0.629	22	11	11	22
332	0.796	0.802	0.836	22	12	22	22
333	0.717	0.651	0.608	22	11	11	12
334	1.046	0.916	0.978	12	11	12	12
335	1.175	1.055	1.122	12	12	12	11
336	0.794	0.763	0.842	22	11	12	12
337	0.875	0.918	1.039	11	12	12	22
338	0.839	0.926	0.748	11	11	22	22
339	0.848	0.668	0.631	22	11	22	11
340	0.645	0.648	0.644	22	12	22	22
341	0.868	0.812	0.761	22	11	12	22
342	0.817	0.715	0.675	22	12	12	11
343	0.838	0.834	0.951	12	11	22	12
344	0.874	0.725	0.761	12	11	12	12
345	0.805	0.703	0.803	22	11	22	11
346	0.869	0.734	0.781	22	11	22	22
347	0.796	0.676	0.761	22	11	22	22
348	0.952	0.77	0.855	22	12	22	22
349	1.025	0.699	0.616	12	11	22	22

350	0.868	0.74	0.859	22	11	22	11
351	0.910	0.667	0.641	22	11	11	11
352	0.930	0.795	0.895	12	11	22	22
353	0.869	0.75	0.768	22	11	12	22
354	0.918	0.875	1.011	22	12	12	22
355	0.932	0.727	0.771	12	12	22	22
356	0.709	0.69	0.682	12	12	22	12
357	0.687	0.735	0.756	22	11	22	12
358	0.928	0.814	0.977	22	11	12	22
359	0.706	0.552	0.576	22	11	22	12
360	1.077	0.96	1.067	12	11	22	11
361	0.792	0.76	0.672	12	11	12	22
362	1.099	0.928	1.063	12	12	12	12
363	0.794	0.763	0.842	22	11	22	22
364	0.747	0.881	0.756	22	11	22	12
