# Supplementary material for: Polymorphisms of FDPS, LRP5, SOST and VKORC1 genes and their relation with osteoporosis in postmenopausal Romanian women
Source: PLoS One. 2019 Nov 27;14(11):e0225776. doi: 10.1371/journal.pone.0225776 (PMC6880991; doi:10.1371/journal.pone.0225776)
Supplement: Attachment — Submitted filename: Response to Reviewers.docx [file pone.0225776.s002.docx]

11.11.2019

Dear Reviewers,

Below you have the responses to the points raised during the review process.

1. Introduction-noted very briefly. Please clarify the source and function of SOST and LRP5 genes in bone remodeling with citations appropriately. For ex., a rationale for genes examined ie anti-resorptive and anti-anabolic. Also, clarify SOST gene product Sclerostin protein is primarily produced by the osteocyte has anti-anabolic effect on bone formation.

Author response: The introduction has been updated accordingly.

“	One of the most important signaling pathways in bone in Wnt, which is crucial for bone development during embryogenesis and has a dual role in bone mass regulation, influencing both bone formation and resorption. The components of the Wnt pathway are proteins involved in cell proliferation, differentiation, and apoptosis of bone cells.[5] When cells are stimulated through the membrane receptors low-density lipoprotein receptor-related protein 5/6 (LRP5/6), the architecture of the multiprotein complex is modified, which inhibits b-catenin, leading to its translocation to the nucleus, where it initiates the transcription of target genes.[6] LRP5 is the most important membrane receptor of the Wnt signaling pathway and it was previously tagged in a genome-wide association study to be associated with OP.[7] LRP5 inactivation caused by mutation is responsible for osteoporosis-pseudoglioma syndrome, in which low bone mass and fractures occur.[8] Also, there are several naturally occurring inhibitors of Wnt signaling, such as Dickkopf (DKK) and sclerostin (SOST) proteins that inactivate signaling from LRP5/6 receptors. Sclerostin, encoded by the SOST gene, antagonizes Wnt signaling in both osteocytes and osteoblasts by binding to the LRP5/6 coreceptor and preventing bone formation.[9.10] High-bone-mass syndromes are thought to be caused by inactivating mutations of SOST (sclerosteosis and van Buchem’s disease).[11] Animal studies have indicated that sclerostin inhibition increases bone mass by stimulating bone formation and inhibiting bone resorption.[12]”

2. Methods-SNP genotyping is described very briefly, and no details of PCR conditions given.

Author response: The PCR conditions has been added to the Material and Methods section

“Genomic DNA was obtained from peripheral blood withdrawn on EDTA, using commercially available kits (Quick gDNA MiniPrep kit, Zymo Research, USA; PureLink Genomic DNA Mini Kit, Invitrogen, Thermo Fisher, USA). We genotyped four SNPs (FDPS rs2297480, LRP5 rs3736228, SOST rs1234612, VKORC1 rs9934438) in all patients and controls using the real-time PCR technique. Standard, predesigned TaqMan SNP genotyping assays, containing all the primers and probes needed for genotyping, were purchased from Thermo Fisher (codes C___2737970_10, C__25752205_10, C___7566033_10, C__30204875_10). All the genotyping were performed according to manufacturer’s instructions. The reaction mix contained 10 μl of 2xTaqMan Genotyping Master Mix (Applied Biosystems, Thermo Fisher, USA), 0.5 μl of the corresponding 40xTaqMan SNP genotyping assay, approximately 25 ng of genomic DNA and free-nucleases water to the final volume of 20 μl. The same amplification program was used for all the genotyping, consisting in a pre-read stage of 30 seconds (s) at 60°C, hold stage of 10 minutes (min) at 95°C, followed by the PCR stage, consisting of 40 cycles, each comprising 15 s at 95°C and 1 min at 60°C, and a post-read stage of 30 s at 60°C. All the experiments were run on a QuantStudio 3 real-time PCR machine (Applied Biosystems, Thermo Fisher, USA).”

3. Results-no rationale given for experiments conducted and this section is noted briefly with no details.

Author response: The rationale behind the experiments have been added

” After dividing the patients into groups, analysis of the codominant model was performed. Codominant models hypothesize that the major allele homozygotes, the heterozygotes or the minor allele homozygotes are associated with the lowest, the intermediate, and the highest risk, respectively, or they are associated with the highest, the intermediate, and the lowest risk, respectively.”

4. References-correct ref#29, follow the journal format ex., remove the month, number in refs etc..

Author response: all references have been modified accordingly.

5. Have the authors made all data underlying the findings in their manuscript fully available? Reviewer response: No

Author response: An Excel file with the raw data has been uploaded with the manuscript files

In the name of all the authors, we thank you for the peer-review process, and we hope the modifications are sufficient and the article is ready to be published in its currents form. If any further clarifications are required, we are happy to give them.

Sincerely,

Dr. Alina Deniza Ciubean
